# Comparative curiosity: How do great apes and children deal with uncertainty?

**DOI:** 10.1371/journal.pone.0285946

**Published:** 2023-05-31

**Authors:** Alejandro Sánchez-Amaro, Federico Rossano

**Affiliations:** 1 Department of Comparative Cultural Psychology, Max Planck Institute for Evolutionary Anthropology, Leipzig, Saxony, Germany; 2 Department of Cognitive Science, University of California San Diego, La Jolla, California, United States of America; Dartmouth College, UNITED STATES

## Abstract

Humans are perhaps the most curious animals on earth, but to what extent our innate motivations for discovering new information are shared with our closest relatives remain poorly understood. To shed light on this question, we presented great apes with two experimental paradigms in which they had to initially choose between an empty opaque cup and a baited opaque cup with rewards invisible to the ape in study 1, or to choose between a transparent cup with rewards or a baited opaque cup with rewards invisible to the ape in studies 2 and 3. We also presented young children with scenarios comparable to the second paradigm (studies 4 and 5). Notably, after the initial choice phase, we presented participants with potential alternatives providing better rewards than the previously secured options. Importantly, those alternatives shared some features with the uncertain options, giving subjects the possibility to relate both options through analogical reasoning. We found that most great apes were not curious about the uncertain options. They only explored those options after they were presented with the alternatives. Children, instead, explored the uncertain options before the alternatives were presented, showing a higher degree of curiosity than the great apes. We argue that differences between children and apes mostly lay in motivational dispositions to explore the unknown.

## Introduction

Animals constantly need to make decisions relevant to their survival and well-being. These decisions are often well informed, i.e. animals are fully aware of their options (e.g., a primate sees food on a tree 30 meters away or a child can choose between two balloons to play with). On other occasions, individuals cannot visualize the consequences of their decisions beforehand but can still infer the probabilities associated with each potential outcome, for instance, by recalling their experiences [1]. For example, chimpanzees can consider multiple factors to plan their next foraging travels to maximize food intake [2, 3] (e.g., a primate learns over years when certain fruits are ripe). In these situations, animals’ decisions are still informed, but they entail risks—they may not get what they aimed for [4–6]. However, it might also be the case that probabilities about future outcomes remain entirely unknown to them. In these situations, animals are uninformed and thus make decisions under uncertainty [7, 8]. Decisions under uncertainty can occur when individuals encounter new objects or scenarios. Imagine, for instance, when a child interacts for the first time with a surprise box. The outcome of the box is unknown.

Nonetheless, children might still be attracted to interact with the box because it may look beautiful, colorful, or simply because they want to discover how it works and the secrets it hides. Children might have a motivation to explore and discover. Some authors have broadly defined this innate drive for information and discovery as curiosity [9–13].

Curiosity may serve to enhance learning by reducing uncertainty [9, 14]. Recent accounts from the developmental literature suggest that children focus more their attention towards stimuli of intermediate complextity over highly predictable stimuli and very complex stimuli, which will require significant cognitive resources [15]. Relatedly, play help children channel their curiosity to reduce uncertainty about the causal structure of the world around them [16]. In addition, the development of curiosity has also been studied using exploration-exploitation dilemmas [17–19]. In these situations, individuals are presented with the possibility to either exploit an already known option or explore other unknown alternatives. Despite the plethora of individual, social and environmental factors that can affect individuals’ decision-making strategies in exploitation-exploration dilemmas, a general finding from the developmental literature suggests that children and adults employ different strategies for exploration. According to Gopnik and colleagues [20, 21], young children are predisposed to sample options more randomly than adults (the cool off effect). However, a recent study by Schulz and collaborators [22] suggested that children reduce their directed exploration as they grow, and children’s exploration becomes more generalizable with age. The authors found that in comparison to adults, children (7 to 11yo) generalized less than adults and relied more often on direct exploration but found no differences in how randomly they explored the environment. The combination of strategies resulted in more but less efficient exploration since the children obtained fewer rewards than the adults (see also [23]).

From an evolutionary perspective, curiosity is undeniably important to help organisms learn about their environment and maximize their fitness. In the context of human evolution, it has been recently hypothesized that high curiosity towards new information was a crucial driver that facilitated cumulative human culture and the early human expansion Out-of-Africa [24]. Interestingly, one of our closest living relatives, the orangutan, usually shows low curiosity levels when presented with novel items unless expert and trustworthy individuals have interacted with the items before [25, 26]. That is when they can make informed decisions after having observed others. In addition, recent studies have found that extensive contact with humans helps orangutans become more curious [27].

However, the concept of curiosity is a hotly debated topic in the cognitive sciences [9, 28]. Curiosity can be understood as a drive to seek information and resolve uncertainty from the world around us, and it seems to be pervasive in humans. From trying new culinary recipes to everyday routines such as checking the news, we constantly look for information to feed our curiosity. Importantly, curiosity in humans is often motivated by internal representations of the need for information [7, 10, 29, 30]. According to Oudeyer and colleagues [31] (see also [32]), only curiosity driven by internal motivations is inherently satisfactory (e.g., the motivation may be the joy of exploring in itself). In contrast, curiosity driven by external motivations involves fulfilling separable outcomes (e.g., the motivation may be related to some potentially achievable result) and not just information for the sake of obtaining it.

In humans, several studies have shown that humans are willing to sacrifice resources in exchange for information for no apparent benefits [14, 33]. Though, whether this internal motivation to resolve uncertainty is shared with other non-human animals is currently debated. In fact, some authors propose that the term curiosity, understood as the drive to seek information *per se*, can only apply when 1) animals sacrifice rewards in exchange for information, 2) the information obtained is not strategically beneficial for the individual and 3) there is a correlation between the amount of resources they are willing to pay and the information available for them [34]. Yet, such strict definition of curiosity is restricted to very specific scenarios [34, 35]. For example, usual tests to determine the role of curiosity in apes problem solving abilities include responses to novel stimuli [36, 37]. However, under the previous definition of curiosity, information obtained from eating a novel food stimuli can be used strategically—they have learned about the food. In addition, their motivation to explore the food in the first place might be extrinsic (a by-product of their exploration tendencies to obtain rewards in the given environment) and they do not need to pay a cost to gain information. Likewise, in uncertainty tasks individuals may only fulfill the first of the three criteria (sacrifice a secure reward to discover another one [8]), but they may then use that information strategically in future events—at the very least, they may have an intuition that the uncertainty option is rewarding as well.

Therefore, given our comparative framework, we deviate from stricter definitions of curiosity (see also [37]) and instead define curiosity as exploration aimed at reducing uncertainty, irrespective of the underlying motivation to do so and the potential future benefits accrued from exploring the unknown. Specifically, we are interested in the moment in which participants decide to sample an uncertain option while foregoing a secure option. This is important because once the uncertain option is sampled, its content is predictable as it does not change across time. That is, although unobservable, participants have the possibility to learn their value over time. In addition, we are interested in exploring whether participants would use strategies such as analogical reasoning (i.e., find a common relationship between two systems, objects, or events [38]) to guide their decisions (see key study details in Table 1).

**Table 1:** Summary of key study differences.

Experimental paradigm	Study	Participants	Research materials	Methodological contingencies
1	1	Great Apes	Opaque cups only /food rewards	Apes had to learn an arbitrary rule to access phase 2
2	2	Great Apes	Opaque and transparent cups /food rewards	Apes had to choose transparent cups in phase 1 to access phase 2 / cups only differed in color
2	3	Great Apes	Opaque and transparent cups /food rewards	No need to choose transparent cups in phase 1/ cups differed in color, material, and shape
2	4	Children	Opaque and transparent cups /stickers	Children received information hints to find stickers/ cups only differed in color
2	5	Children	Opaque and transparent cups /stickers	No information hint/ cups differed in color, material, and shape

For that purpose, the current study present captive non-human great apes and 3- to 5yo children with two distinct experimental paradigms across five experimental studies.

In our first experimental paradigm (study 1), great apes learned to discriminate between two different colored cups [39]. Only one of the colors signaled a reward. After learning the perceptual association, individuals participated in a short priming phase where they associated new colors with either more rewards or zero rewards. Finally, great apes participated in two test sessions, including trials in which they had to decide between the initial colored cup with a reward and a new positive or negative color association. We expected that only those individuals that had previously experienced the positive priming would be willing to explore new ambiguous possibilities.

The previous experimental paradigm required individuals to learn an arbitrary rule based on color. In line with our first study, previous studies show that apes take several sessions of multiple trials to learn the relationship [39–41]. Studies 2 and 3 facilitated the task by presenting great apes with a decision between an opaque cup containing three unknown rewards and a transparent cup containing only one reward. Afterward, individuals participated in an experience phase to obtain three rewards underneath a different type of opaque cup. Finally, apes were again presented a decision between the initial set of transparent and opaque cups. Previous work has found that apes effectively use relational similarity concerning the spatial location of an object, but not so often object similarity in a searching task [42]. We hypothesized that apes would use relational similarity to explore the colored cup significantly more often after the experience phase. In other words, through analogical reasoning, they would become more willing to explore the content underneath the initial opaque cup (see Table 1 for differences between studies 2 and 3).

Studies 4 and 5 use the previous paradigm to test children’s curiosity under conditions of uncertainty. We expected children to choose the opaque cup more often before the experience phase compared to great apes. In other words, we expected children to prefer the opaque cup in uninformed situations already (see Table 1 for differences between studies 4 and 5). Finally, given their similarity, we directly compared the performance of apes and children in studies 3 and 5. To our knowledge, no studies have directly contrasted children’s and great apes’ uncertainty reduction tendencies in comparable experimental settings.

## Methods great ape studies

### Study 1

#### Participants

We tested 29 great apes: 15 chimpanzees (10 females; mean age 30 years old), three gorillas (3 females; mean age 14 years old), six bonobos (4 females; mean age 22 years old), and five orangutans (3 females; mean age 26 years old) (see S1 Table in S1 File). The following details apply to studies 1–3. All apes were housed at the Wolfgang Köhler Primate Research Center in Leipzig (Germany). Chimpanzees were housed in large semi-natural indoor and outdoor enclosures and the research was conducted in their sleeping rooms. Apes had regular feeding schedules, daily enrichments and water ad libitum. Apes were never food- or water-deprived and could voluntarily participate in the test by entering their sleeping rooms. During the test sessions chimpanzees had access to water ad libitum. After the test they return to their enclosures and reunite with the groupmates.

#### Materials

The apparatus consisted of a rectangular platform (78 x 33 cm) attached to a Plexiglass panel (73 x 64 cm) installed on the front side of the apes’ sleeping room. The platform could be slid forward against the panel. The panel had two equidistant holes in the opposite bottom corners (60 cm apart, 3.2 cm diameter). We used varying quantities of grapes as a food reward and thin plastic cups of diverse colors to cover the food rewards. Besides, we used a plastic lid (approx. 75 cm wide, 25cm deep, and 25 cm tall) to cover the surface of the platform from the apes’ view while the experimenter hid the food rewards underneath the cups.

#### Procedure

At the beginning of a trial, the experimenter (hence E) sat in front of the sliding platform and covered it with a plastic lid. Next, E baited some grapes on the sliding platform and covered them upside down with the plastic cups. The number of grapes varied depending on the condition presented. Afterward, E moved the plastic cups towards the edges of the sliding platform—each cup in front of a panel hole. E then removed the plastic lid and pushed the sliding platform towards the Plexiglas panel. We considered a choice when the ape touched one of the two cups, pointed at it, or put her mouth next to the hole. If the subject chose more than one cup simultaneously, E pulled the sliding platform back and repeated the procedure. After the ape chose one of the cups, E uncovered the food under the cup and handed it to the ape through the panel hole. The content baited under the unchosen cup was uncovered in some of the conditions presented (see below). After the subject obtained the reward, E pulled back the platform and prepared for the subsequent trial.

**Fig 1:**
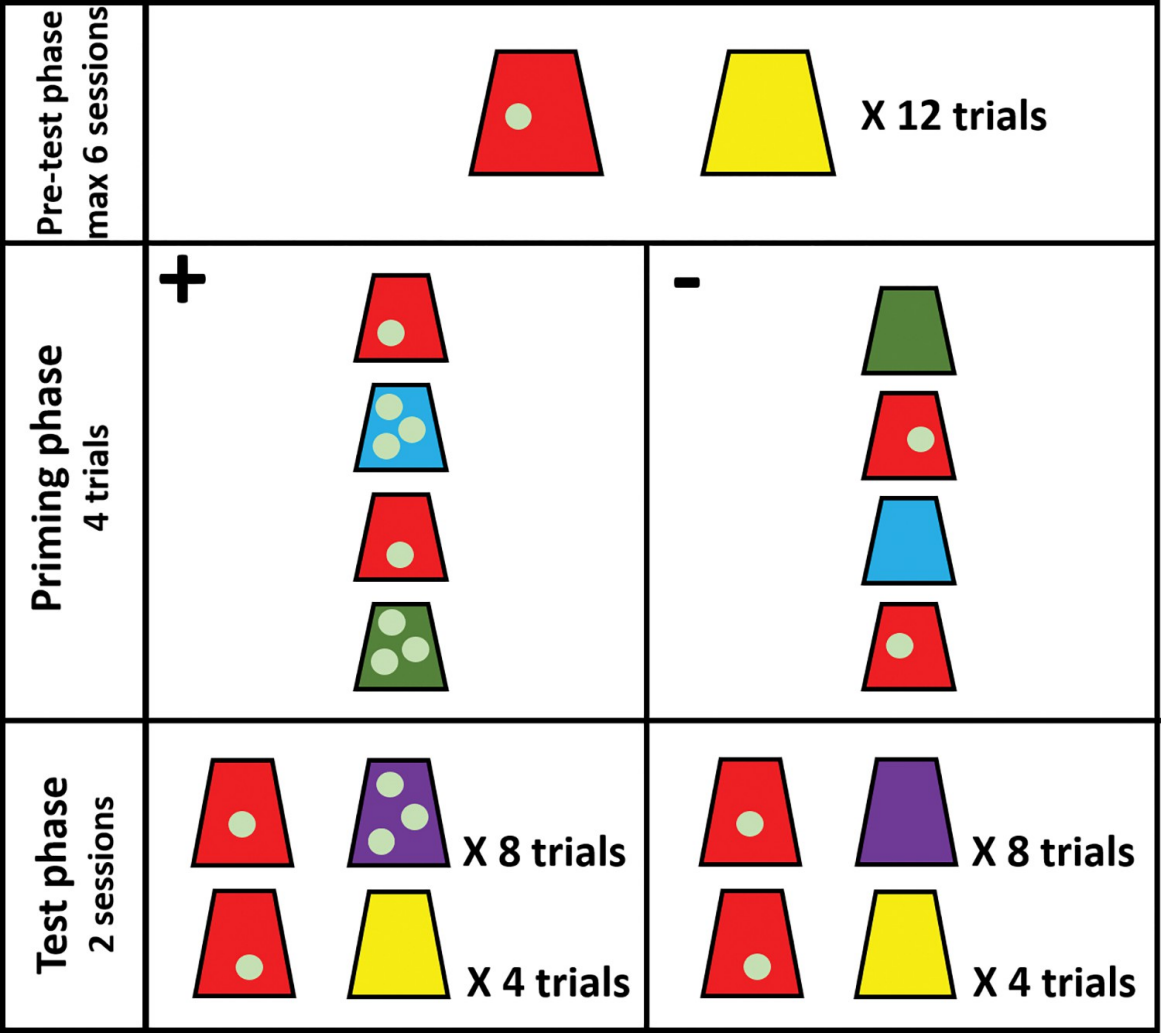
Schematic representation of study 1 conditions.

The study was divided into three phases (see Fig 1). During the pre-test phase, each ape participated in a maximum of six 12-trial sessions on separate days. Apes that were successful during the pre-test phase participated in the priming and the test phases. The priming phase and the first test session were conducted on the same test day. The second test session was conducted on a separate day.

#### Phase 1: Pre-test phase

During the pre-test phase, apes had to learn the association between the color of the cup and the hidden reward. In every trial, apes could choose one of two plastic cups. These plastic cups were identical except for their color (e.g., red vs. yellow). Only one of the two cups was baited with a single grape. Importantly, we always baited the same colored cup. This way, the color of the cup indicated the presence or absence of a reward (e.g., the red cup was baited with a grape and the yellow cup was empty). Each cup was located in front of a panel hole on either side of the sliding table. The location of the cups was pseudo-randomized between trials. The location of the cups was not repeated for more than two consecutive trials within a session. Apes had to choose the baited cup in at least 10 of 12 trials for two consecutive sessions to advance to the study’s second phase. After the apes chose one of the cups, the content of both cups was revealed at the same time. Only eight great apes (four chimpanzees, two bonobos, one orangutan, and one gorilla) advanced to the priming phase (see S1 Table in S1 File). All other apes did not reach the criteria within six sessions.

#### Phase 2: Priming phase

The priming phase consisted of one single session of four trials. On every trial, the subjects were presented with a single colored cup on the left or the right side of the sliding table. In two trials, apes were presented with the *previous positive cup* (PPC) used during the pre-test phase (e.g., the red cup baited with one grape). In the other two trials, the apes were presented with two new colored cups (e.g., one trial with a green cup and another with a blue cup). These cups were either empty (negative priming) or baited with three grapes (positive priming). The location of the cups varied pseudo-randomly—the apes faced each type of cup only once on each side of the table. Four subjects experienced the positive priming, and another four experienced the negative priming.

#### Phase 3: Test phase

The test phase consisted of two 12-trial sessions. As in the pre-test phase, apes had to choose one of two different colored cups on each trial. In eight test trials, apes faced the PPC (e.g., the red cup with one grape) and a completely new colored cup they had never interacted before in the experiment (e.g., a purple cup). Depending on whether the subject experienced a positive or a negative priming, the new cup could either be baited with three grapes (after the positive priming) or empty (after the negative priming). This cup remained the same during the test phase. In addition, all apes faced four control trials that were the same as those presented during the pre-test phase. As in the pre-test phase, the location of the cups was pseudo-randomized. After the apes chose one cup, the content of the other cup was never revealed.

### Study 2

#### Participants

We tested 15 captive great apes: 9 chimpanzees (5 females; Mean age 30yo) and six bonobos (4 females; Mean age 22yo) housed at the Wolfgang Köhler Primate Research Center in Leipzig, Germany (See S1 Table in S1 File).

#### Materials and procedure

The apparatus and trial presentation were the same as in study 1. However, the study phases differed from the previous paradigm. In particular, apes did not need to learn an arbitrary rule to obtain the reward (i.e., the color of the rewarded cup). In contrast, they always chose between an opaque colored cup containing unknown rewards and a transparent cup containing a single reward.

**Fig 2:**
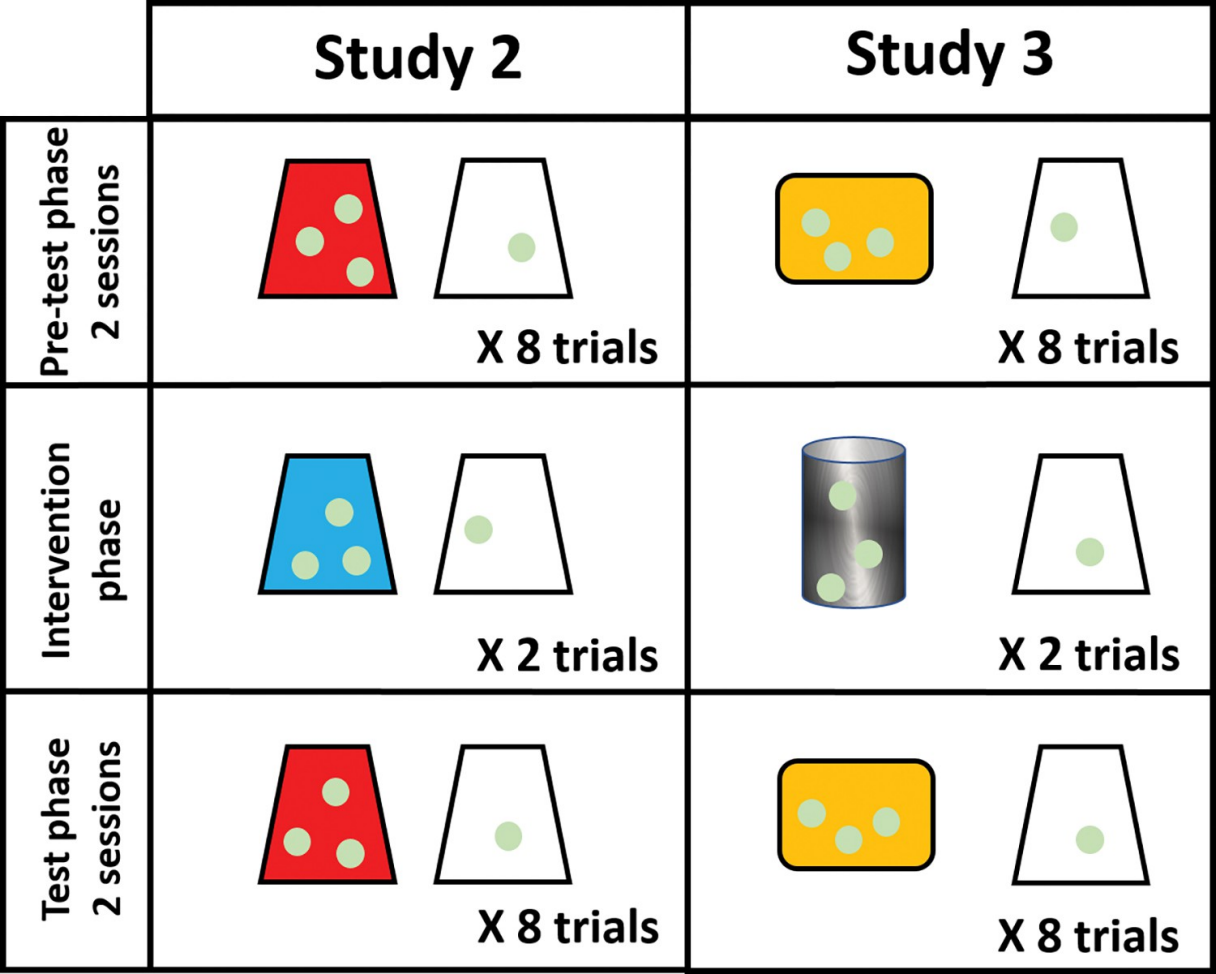
Schematic representation of study 2 conditions. On the right side, schematic representation of objects used in study 3.

The study was divided into three phases (see Fig 2). First, every ape received two 8-trial pre-test sessions (phase 1). Afterward, apes participated in two intervention trials in which they were presented with the same transparent cup and another opaque cup (phase 2). Opaque cups of phases 1 and 2 only differed in their color. Finally, apes conducted two 8-trial test sessions (phase 3). Phase 1 and phase 3 were identical in terms of conditions and rewards. We conducted Phase 2 and the first test session of phase 3 on the same test day.

#### Phase 1: Pre-test phase

The study’s first phase consisted of two 8-trial pre-test sessions presented on separate days. In every trial, we presented apes with the option to choose one of two plastic cups: a transparent or an opaque cup. The opaque cup (e.g., red cup) was baited with three grape halves, while the transparent cup was baited with one half of a grape. Each cup was located in front of a panel hole on either side of the sliding platform. The location of the cups was pseudo-randomized between trials. In other words, the location of the cups was not the same for more than two consecutive trials within a session, and each cup was presented the same number of times on each side of the platform. Subjects had to choose the transparent cup on every trial to demonstrate a clear preference and thus advance to the study’s second phase. The content of the opaque cup was never revealed to the subjects after choosing the transparent cup.

#### Phase 2: Intervention phase

The second phase of the study consisted of two trials in which apes were offered a choice between the same transparent cup presented in phase 1 and another opaque cup of a different color from the one used in phase 1 (e.g., a blue cup). The moment the apes were deciding on their preferred cup (i.e., while deliberating which cup to choose), E revealed the content hidden under the opaque cup twice. This action allowed apes to reconsider their choice. If apes did not change their decisions, they got the reward from their chosen cup. If apes changed their decision, they got the reward from their last chosen cup. The location of the cups varied between trials.

#### Phase 3: Test phase

The third phase of the study was identical to the first phase. We used the same opaque cup in phases one and three.

### Study 3

#### Participants

We tested 21 captive great apes: 11 chimpanzees (6 females; Mean age 26yo), four bonobos (2 females; Mean age 22yo) and 6 orangutans (4 females; Mean age 25yo) housed at the Wolfgang Köhler Primate Research Center in Leipzig, Germany (see S1 Table in S1 File).

#### Materials and procedure

In study 2, only those apes who always chose the transparent cup during phase 1 were allowed to participate in phases 2 and 3. Accordingly, only a subset of apes participated in all the study phases. In study 3, we followed the same procedure as study 2, but we tested all participants across the three phases regardless of their choices during phase 1. We think such an approach is more inclusive and representative of the apes’ initial preferences compared to the approach used in study 2. In addition, we made some minor adjustments to the methodology of the second study.

In study 3, the cups did not just differ in color as in study 2, but also in material and shape (see Fig 2). Transparent cups were also partially colored (although the color did not prevent apes from seeing the rewards baited underneath). We manipulated these features for two main reasons. First, apes could have interpreted all colored cups as the same type of cups in study 2. To increase the differences between cups, we thus used items varying in more than one perceptual feature. Second, we used different items to avoid order effects, given that a subset of those individuals who participated in study 2 until the end also participated in study 3 several months later (10 of 21 apes). In addition, we varied the number of rewards from study 2 to diminish potential order effects affecting some apes’ performance. We placed two grapes underneath the opaque cups and one grape underneath the transparent cup in the third study. Finally, in phase 2 of study 3, we revealed the content of the opaque cup after the subjects had made a clear decision. We changed the method because, in study 2, it was sometimes difficult to see what the apes had decided without touching the cups.

## Methods children studies

### Study 4

#### Participants

We tested 72 3- to 5yo children. All children were tested in their local preschools or social institutions such as aquariums or museums in the San Diego County in the United States. We grouped children by age (3, 4, and 5yo) and gender, resulting in 24 children per age group (12 girls and 12 boys). For studies 4 and 5, children were only recruited for participation after written consent from their parents or legal guardians.

#### Materials

We used stickers as rewards. Stickers were hidden under cups placed on a table. The cups were either transparent or opaque, and the latter varied in their color. The cups were similar to those used in study 2 with the apes. We used a plastic clipboard (48 cm wide X 30 cm tall) to cover the cups while the experimenter hid the corresponding stickers underneath them. The child sat on a chair in front of the table. The experimenter sat in front of the child on the opposite side of the table.

#### Procedure

At the beginning of the session, the experimenter (E) told the child that he/she would participate in a game to find stickers and that the stickers would be hidden under plastic cups—while briefly showing the cups they would use in phases 1 and 3. E also told the child to point to the cup he or she had chosen on every trial. Besides, E gave an additional plastic cup to every child to store the collected stickers.

At the beginning of every trial, E used the clipboard to cover the cups in front of the child. Next, E placed some stickers on the table and covered them with the plastic cups upside down. E placed one plastic cup on the right side and the other on the left side of the surface covered by the clipboard (approx. 10 cm apart from each other). E then removed the clipboard and waited for the child to point to a cup. E reminded her to choose a cup if the child did not point. If the child tried to grab the cup, E tried to stop the child and remind her to point to a cup. We considered a choice when the child pointed or touched one cup. After the child chose a cup, E gave the child the content under the selected cup and prepared the subsequent trial.

Every child received one session of 10 trials divided into three phases. First, children were presented with four consecutive pre-test trials (phase 1). Next, they were presented with two trials (phase 2) in which they were confronted with the same transparent cup and another opaque cup of a different color. Finally, children conducted four test trials (phase 3) identical to those presented in phase 1.

#### Phase 1: Pre-test phase

The first phase of the study consisted of four consecutive pre-test trials. In every trial, children were presented with a choice between an opaque and a transparent plastic cup. The colored cup was baited with three stickers, while the transparent cup was baited with one sticker. Cups were presented in a pseudo-randomized order. The location of the cups was not repeated for more than two consecutive trials, and each cup was presented twice on each side of the table. The content of the opaque cup was never revealed to the subjects after choosing the transparent cup.

#### Phase 2: Intervention phase

The second phase of the study consisted of just two trials. Children were offered a choice between the transparent cup presented in phase 1 and another opaque cup of a different color. The transparent cup contained one sticker, and the opaque cup contained three stickers, as in the previous phase. If children chose the transparent cup, E revealed the content of the opaque cup. At that moment, children were allowed to choose again with the possibility to change their choices if they wished. The location of the cups varied between the two trials.

#### Phase 3: Test phase

The third phase was identical to phase 1, and the opaque cup was the same one we used in phase 1. After phase 3, children were allowed to choose three stickers among those collected during the game. Children were never told to choose a number of stickers before that moment. In that sense, it is very likely that their expectation was to collect and keep all the stickers they discovered.

### Study 5

#### Participants

We tested 52 3- and 5yo children. All children were tested in their local preschools or in social institutions such as aquariums or museums in the San Diego County in the United States. We grouped children by age and gender (12 3yo boys, 15 3yo girls, 12 5yo boys, and 13 5yo girls).

#### Materials and procedure

In study 4, we told children that the game’s purpose was to find stickers. This information could have increased children’s likelihood of exploring all possible alternatives from the onset of the study, including the opaque cups. Additionally, the fact that children had more information than great apes from the onset of the task could hinder comparisons between species. Thus, to keep our methods consistent with the studies conducted with the great apes (studies 2 and 3 especially), in study 5, we reduced the explanations children received prior to the task. We told the children that they would play a game "about" stickers and that they should always choose one of the two cups. In addition, we made some minor improvements to the methodology of study 4.

In study 5, we used stickers of the same type across trials of a session to control for size and quality (i.e., emojis with different facial expressions). In the previous study, we showed children the content of the opaque cup either during or after they chose any of the two cups, allowing them to reconsider their decisions and change their choices. This reconsideration could have been interpreted as a mistake from the children’s perspective and could have influenced their subsequent decisions. Therefore, in phase 2 of study 5, we showed children the content of both cups before they chose one of the two. Finally, to make our results more comparable to great apes, we presented children with cups varying in color, shape, and material.

## Ethics

An internal ethics committee approved studies 1–3 at the Max Planck Institute for Evolutionary Anthropology. The study complies with the Weatherfall report ‘The use of non-human primates in research’. The study also complies with the EAZA Minimum Standards for the Accommodation and Care of Animals in Zoos and Aquaria, the WAZA Ethical Guidelines for the Conduct of Research on Animals by Zoos and Aquariums and the ASAB/ABS’s Guidelines for the Treatment of Animals in Behavioural Research and Teaching. IAUCUC approval was not necessary to conduct this research.

The work conducted with children in studies 4 and 5 was approved by the University of California, San Diego Human Research Protections Program (Project ID: #161452SX).

## Results

### Study 1

**Fig 3:**
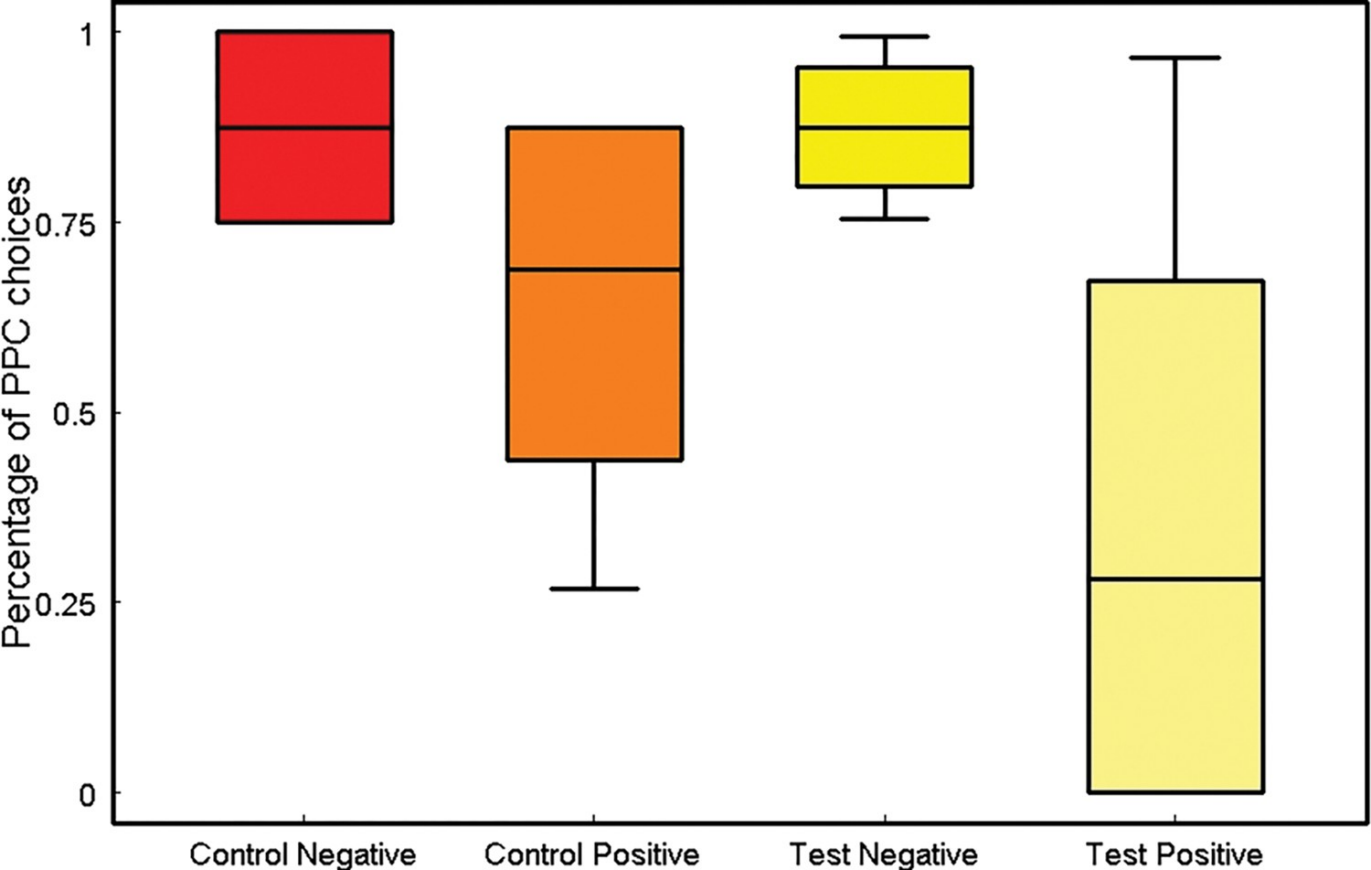
Percentage of trials in which apes chose the PPC option after control and test trials and positive or negative primes. The boxes represent the median and the interquartile range.

In this study, we were interested in exploring how often the apes would refuse the PPC during test trials based on the priming they had experienced before the test phase. As hypothesized, our Generalized Linear Mixed Model (GLMM) found a main effect of the previous priming phase indicating that those great apes who had experienced positive priming were more likely to refuse the PPC in favor of the new ambiguous option. However, these apes were more likely to choose the cup containing no rewards during control trials (GLMM: *χ*^2^_1_ = 10.77, *p* = 0.001, N = 192, Fig 3), thus missing the opportunity to obtain the hidden grape under the PPC. We argue that apes might have generalized the idea that alternatives were better than the PPC, which could explain their poorer performance in control trials after the positive priming. Nevertheless, they still chose the PPC in most control trials (see Fig 3). In contrast, apes who experienced negative priming were more likely to continue choosing the PPC in test and control trials alike (see model details in the S1 File).

### Study 2

In our second study, we expected apes to prefer the transparent cup initially, and to become more curious to explore the opaque cup after the intervention phase. In line with our predictions, most apes always chose the transparent cup in phase 1 (Binomial test; 11 of 15; p = 0.06). We found that those subjects who always chose the transparent cup in phase 1 dramatically changed their choices between phases 1 and 3 (Wilcoxon paired sample test, *p* < 0.01). All but one individual chose the opaque cup most times during phase 3 (average opaque choice in phase 3 = 86%). The apes chose the opaque cup in 100% of the trials during the intervention phase.

### Study 3

As in study 2, in our third study, we were interested in whether apes would vary their choices between phases 1 and 3 (i.e., before and after the intervention phase). In other words, we assessed whether apes would become more willing to explore the uncertain option after the intervention phase, especially in cases where they had a clear preference for the transparent phase. We found that, as in study 2, a significant majority of apes only chose the transparent cup in phase 1 (Binomial test; 16 of 21; *p* = 0.015). However, in contrast to study 2, in study 3 all apes participated in the subsequent phases of the study. We found that apes significantly changed their choices between phases 1 and 3 (Wilcoxon paired sample test, *p* <0.001) (average opaque choice in phase 1 = 16%; average opaque choice in phase 3 = 68%). The apes chose the opaque cup in 90.4% of trials during the intervention phase.

**Fig 4:**
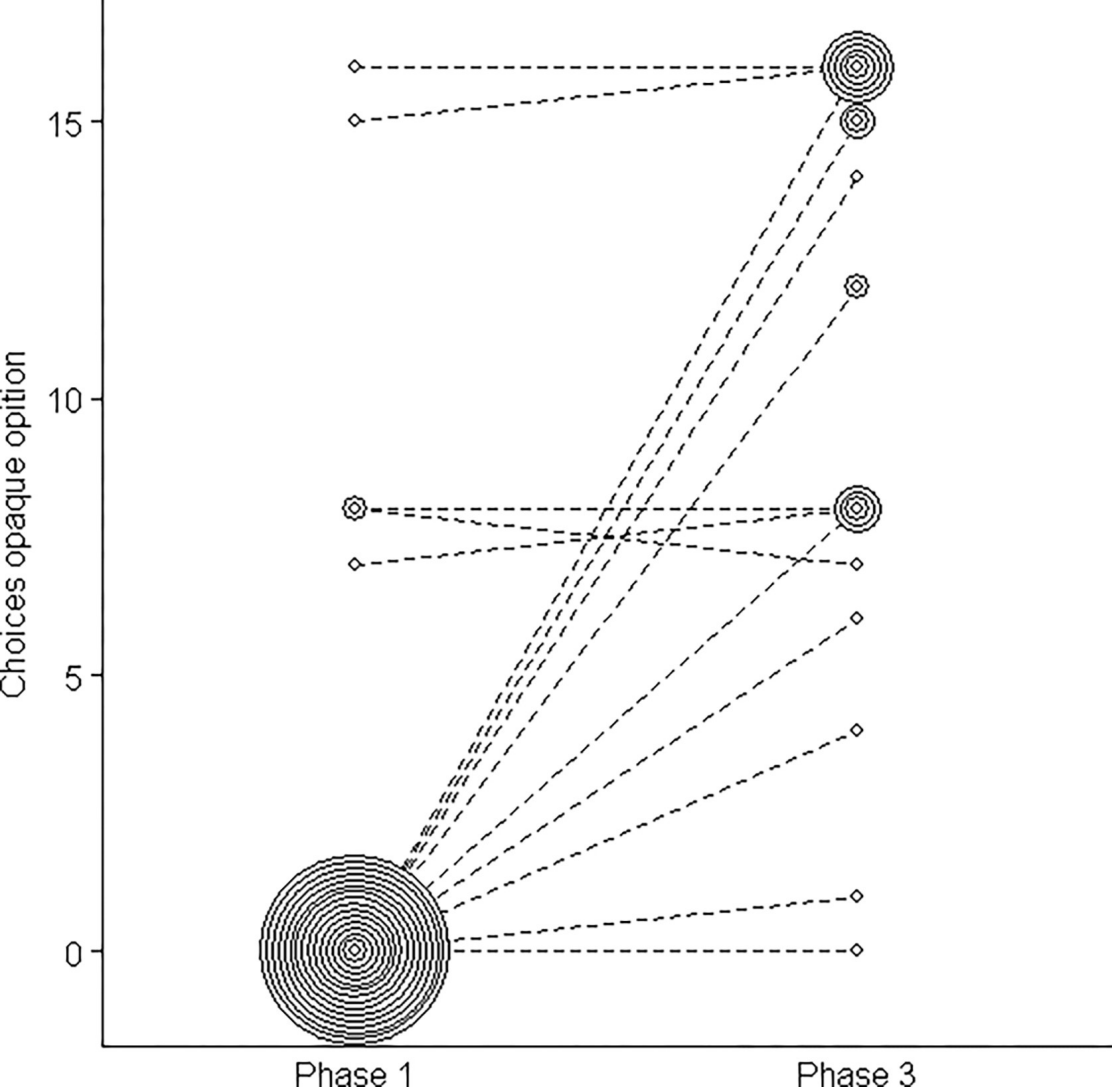
Bubble plot depicting the number of times each ape chose the opaque option in phases 1 and 3.

In addition, we fitted a GLMM to test whether great apes’ decisions (to choose the opaque or the transparent cup) were influenced by the study phase and whether there were species differences. We found that the full-null model comparison was highly significant (GLMM; *χ*^2^_3_ = 16.23, *p* = 0.001, N = 672). Great apes chose the opaque cup significantly more often in phase 3 (GLMM; *χ*^2^_1_ = 11.49, *p* = 0.001, N = 672, Fig 4) but we found no differences between species (GLMM; *χ*^2^_2_ = 2.57, *p* = 0.28, N = 672) (see model details in the S1 File). Interestingly, two apes chose the opaque rewards a majority of times in study 2. These two individuals had participated in study 2, and thus order effects could have influenced their performance in the task. Three other apes showed side biases markedly. Consequently, they chose the opaque option around half of the time across phases.

### Study 4

In our first children’s study, we wanted to investigate whether discovering other positive alternatives would influence their choices during the third study phase or if, as we hypothesized, children would be more prone to choose the opaque cup already during the first study phase. In line with our hypothesis we found that most children chose at least once the opaque cup in phase 1 (Binomial test; 61 of 72; *p* < 0.001). In general, children were curious to explore the content underneath the opaque cup from the onset of the study (average opaque choice in phase 1 = 52%; average opaque choice in phase 3 = 77%). Children chose the opaque cup in 85.4% of trials during the intervention phase. In another 20.1% of trials they originally chose the transparent but switch to the opaque after we revealed the content of it. Furthermore, once children had experience the content of the opaque cup, they continued choosing the transparent cup in 27% of the following trials on average.

As with the apes’ data, we fitted a GLMM to test whether children’s decisions to choose the opaque or the transparent cup were influenced by the study phase and whether older children were more curious about the content of the opaque cup. We found that the full-null model comparison was highly significant (GLMM; *χ*^2^_3_ = 12.17, *p* = 0.007, N = 574). Children were more likely to choose the opaque cup in phase 3 (GLMM; *χ*^2^_1_ = 7.43, *p* = 0.006, N = 574, S1 Fig in S1 File). In addition, we found a non-significant trend for age suggesting that children increased their choices towards the opaque cup with age (GLMM; *χ*^2^_2_ = 4.5, *p* = 0.1, N = 574, S2 Fig in S1 File) (see model details in the S1 File).

#### Study 5

In the previous study, children were instructed to find stickers. In this study, we did not prime them with that information. Therefore, we expected children to still explore the uncertain option prior to the intervention, but at lower rates compared to study four. Most children chose at least once the opaque cup in phase 1 (Binomial test; 40 of 52; *p* < 0.001). However, we found more children sticking to the transparent option in phase 1 of study 5 compared to phase 1 of study 4. In addition, several children (40%) made the same choices across phases (average opaque choice in phase 1 = 41%; average opaque choice in phase 3 = 56%). Children chose the opaque cup in 69% of trials during the intervention phase. Furthermore, after experiencing the opaque option for the first time, children still chose the transparent cup in 47% of the following trials on average.

**Fig 5:**
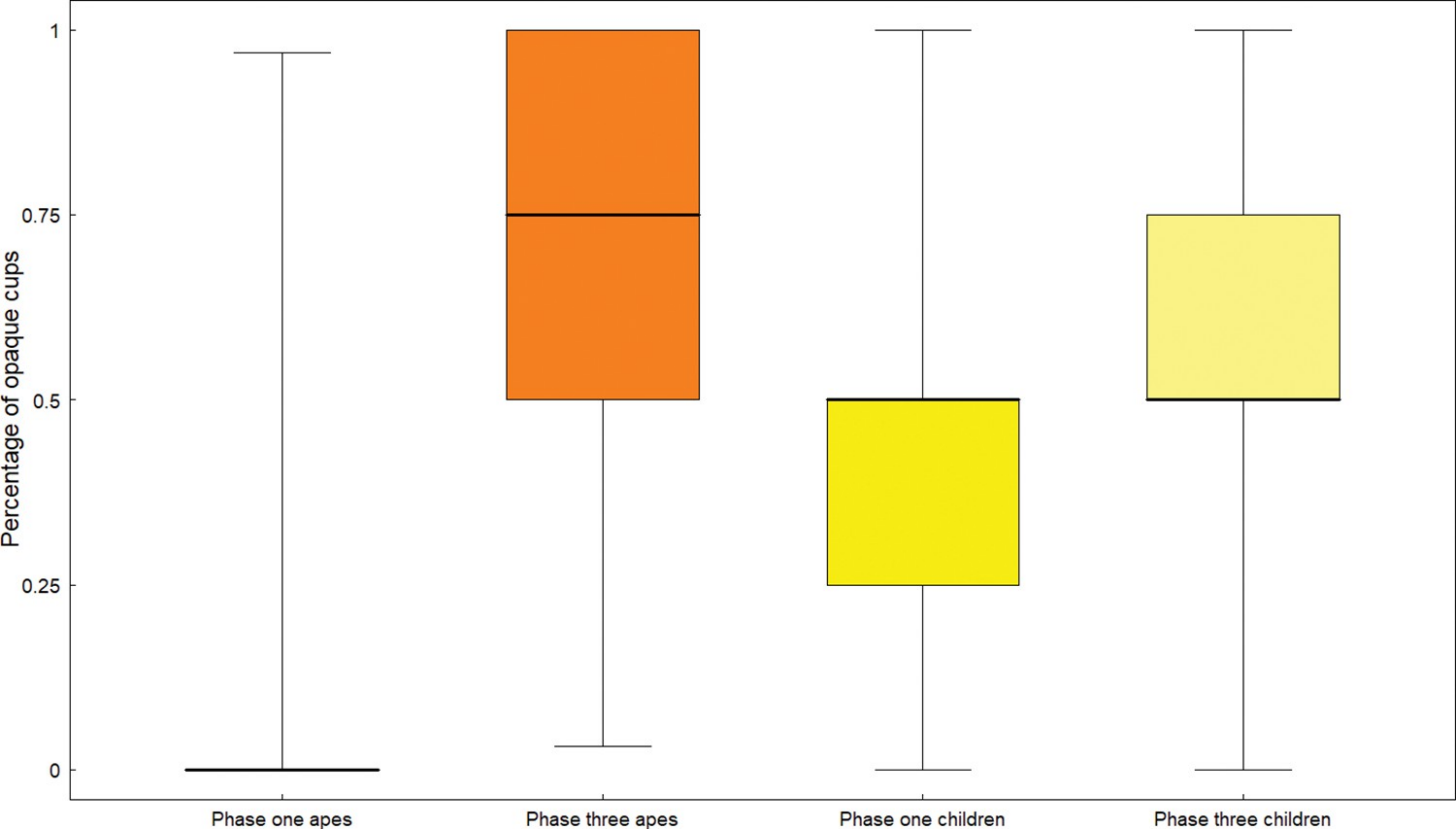
Bubble plot depicting the number of times each child chose the opaque option in phases 1 and 3.

We fitted a GLMM to test whether children’s decisions to choose the opaque or the transparent cup were influenced by the study phase and whether older children were more curious about the content of the opaque cup. We controlled for learning effects (trial) and sex of the participants (see model details in S1 File). We found that the full-null model was significant (GLMM; *χ*^2^_3_ = 6.07, *p* = 0.048, N = 415). Children were more likely to choose the opaque cup in phase 3 (GLMM; *χ*^2^_1_ = 4.42, *p* = 0.035, N = 574; Fig 5). We did not find an age effect suggesting that 3- and 5yo explored in similar ways.

### Direct comparison of studies 3 and 5

**Fig 6:**
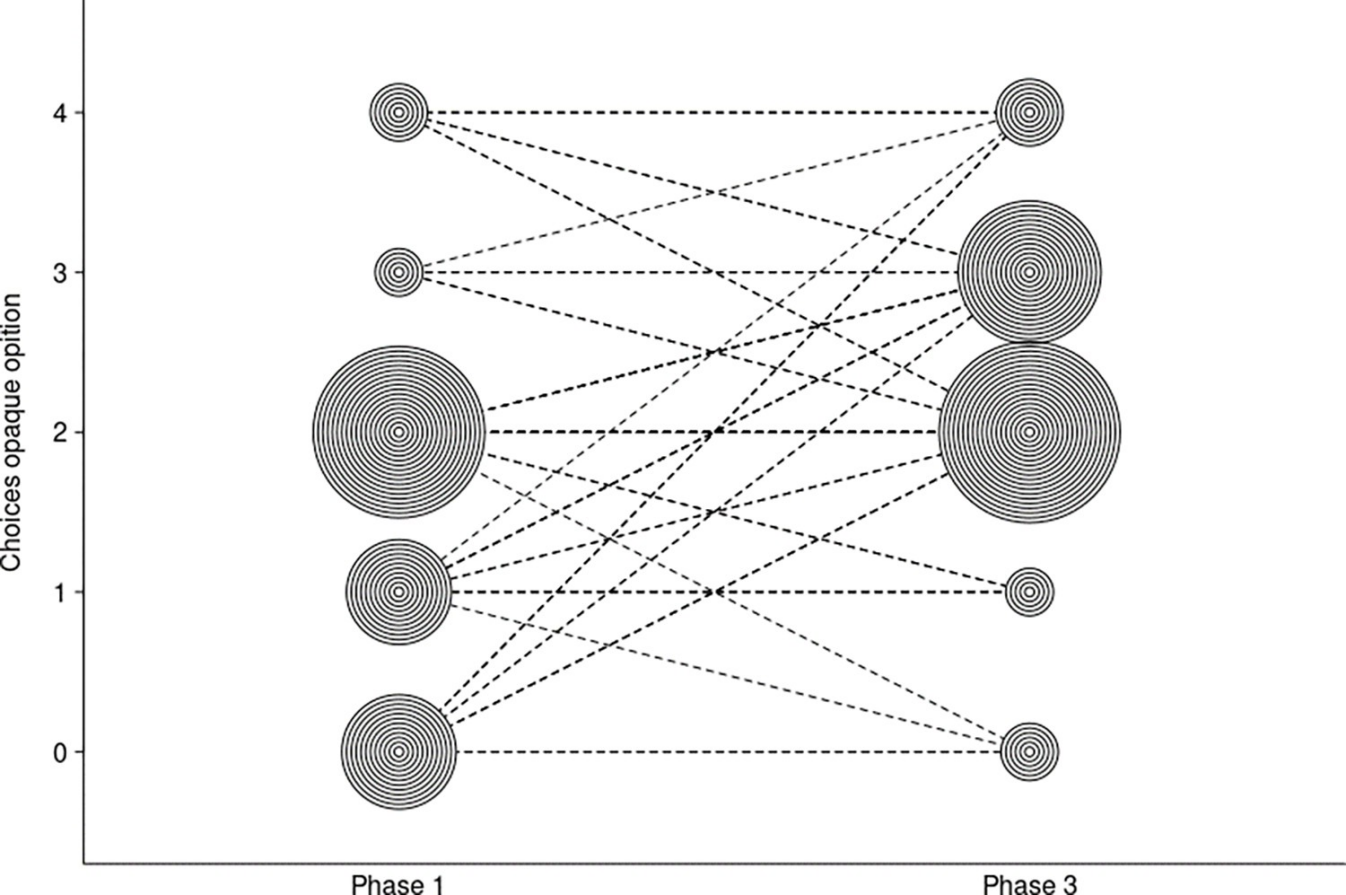
Percentage of trials in which apes and human children chose the opaque cup in phases 1 and 3 of studies 3 (apes) and 5 (children). The boxes represent the median and the interquartile range.

Additionally, we fitted a GLMM to test whether children and great ape decisions to choose the opaque or transparent cups differed. In our model we tested the interaction between species (“apes” and “human children”) and study phase. We controlled for trials and sex of participants as in previous models (see model details in S1 File). We found a significant two-way interaction between specie and study phase (GLMM; *χ*^2^_1_ = 8.72, *p* = 0.003, N = 1087). Children were more likely to explore the opaque option in the pre-test phase while apes mostly chose the opaque option after the priming phase (see Fig 6).

## Discussion

Our set of five studies revealed two main findings. First, children seemed to be more willing to explore the uncertain cup than apes in similar test situations (studies 2 and 3 vs. studies 4 and 5). While apes mainly chose the transparent cup during phase 1 of studies 2 and 3, most children chose the opaque cup during the first phase of studies 4 and 5. This difference is of particular interest in study 5, in which children were never told to find rewards—thus resembling more closely the scenario the apes experienced. Second, we found that children and apes became more explorative across studies once they had experienced the intervention phase. In study 1, apes who had experienced the positive prime were more likely to choose the ambiguous option during the test phase than those who experienced the negative prime. Likewise, in studies 2 to 5, children and apes became more explorative after the intervention phase.

Therefore, one intriguing question from our findings is why were children generally more willing to choose the opaque options before the intervention phase than great apes?

We would argue that differences between children and apes mostly lay in motivational dispositions to explore the unknown. A predisposition to discover what was hidden underneath the opaque options could have driven children, but not great apes, to select opaque cups before the intervention phase. From the beginning of the task, curiosity could have been influenced by external factors such as the prospect of increasing rewards or by internal motivations to reduce uncertainty [31, 32]. This distinction could help explain the differences between studies 4 and 5. The former study explicitly told children about the game’s goal, while in the latter the strategic gains (and thus external factors) from choosing the opaque cup only became clear once they chose that option. Another plausible interpretation is that apes were more risk averse than children from the beginning of the study, and only felt more confident to explore the unknown possibilities after the intervention phase—when they changed their strategy favoring the selection of the opaque cups.

Consequently, children were more willing to explore the opaque cup before the intervention phase during study 4 than during study 5, and also maximized more the rewards during the test phase of study 4 compared to study 5 suggesting that when children were provided with information about the goal of the task, their decisions were more directed. Instead, children’s exploration was more random when they had no information about the game’s purpose [43]. Nevertheless, in study 5, most children still explored at least once the opaque option before the intervention phase, suggesting that, overall, internal motivations strongly influenced children’s decisions.

In any case, children were not always willing to forego secure rewards. Many children continued to choose the transparent cup even after they had experienced the content underneath the opaque cup. This was especially the case in study 5 when they had never been prompted to find stickers. In fact, children continued to choose the transparent cup in almost half of the trials after they had experienced the content of one opaque cup. Several possibilities may account for this result. Perhaps children did not believe that each opaque cup yielded better rewards than transparent cups. After all, there was always some uncertainty in choosing the opaque cup. It is also possible that children preferred to diversify rather than maximize their outcomes. Such a strategy could explain some of the findings in study 4 since all stickers were different. In study 5, however, stickers’ variation was drastically reduced (no differences in size or shape). Another possibility is that children preferred to play by their own rules: trying to guess if some order of events was in place or simply preferring to choose differently across trials.

Apes, in contrast, seemed to avoid the opaque cups from the beginning of the study. This lack of exploration may be understood as an aversion toward uncertainty [8, 44]. Under uncertain situations, individuals do not have prior information about any probability of obtaining a benefit. Therefore, the opaque cup could have been interpreted as a potential loss in contrast to the visible and rewarding cup at the beginning of the task. Even though they knew that the two options were available to choose from, they seldom chose the opaque cup until its content was revealed during the intervention phase. An alternative is that apes did not interpret the opaque cup as an option. However, we do not favour this interpretation. The apes from this population are used to participate in choice scenarios where food rewards might be completely or partially covered by an opaque object (see [45] for a recent longitudinal task conducted on the same apes employing opaque cups in some of their tasks).

Interestingly, the realization that the opaque alternatives presented during the intervention phase offered them more rewards than the transparent cups helped many apes overcome such an initial risk aversion toward uncertain choices quickly. That is, apes generalized from just a couple of trial presentations during the intervention phase to the content hidden in the test trials—regardless of how different the items between the two phases were. We argue that this generalization could not be driven by simple association. Previous tasks using arbitrary color cues demonstrate that apes need several multi trial sessions to learn the relationship (as in the pre-test of our first study) [39–41]. Instead, we argue that this generalization was most likely supported by processes of analogical reasoning [38, 42] through which apes established relational similarities between distinct stimuli, allowing them to maximize rewards across tasks. The same argument can be used for the results in study 1. Apes established relational similarities between the cups presented during the intervention phase and the new colored cups presented during the test phase. However, the apes’ results contrast with what many children did after exploring the opaque cups. Children often chose the transparent cup during the test phase. This strategy mainly occurred during study 5, where no search clue incentives were provided, supporting the view that children might have employed diverse strategies other than trying to maximize the number of rewards in every trial.

One could also propose that inhibitory control differences underlie children and great apes’ strategies in our tasks. However, we argue that differences in inhibitory control cannot explain our results. First of all, apes and children were able to inhibit a prepotent response towards the visible rewards. In that sense, both groups showed inhibition towards the direct reward, which helped them explore the uncertain cup and discover the hidden rewards. For study 1, inhibitory control does not seem a relevant explanation either since both cups were occluded from the beginning of the pre-test phase. Instead, these inhibitory control capacities are relevant in reverse contingency tasks where individuals must overcome a prepotent preference toward a high-value reward. They need to exert self-control to inhibit their initial preference and choose the less valuable option to obtain the best reward [39, 46, 47]. However, that was not the case in our tasks since only one reward was visible and known (or known after a considerable training period as in study 1). It is also worth highlighting that previous comparative work showed that when 3-to-5yo children and great apes were presented with a detour reaching task, there were no practical differences between the 3yo and the same chimpanzee population that participated in our tasks [48].

Future studies can extend these paradigms in several ways. First, the apes who participated in our task were familiarized with the stimuli we used (e.g., plastic covers or wooden boxes). On the one hand, this casts doubt on the possibility that novelty explains the pattern of results observed. In addition, familiarity with human-made items facilitated the comparison between apes and children. However, one could argue that the tasks were still more ecologically relevant for children, who might be more used to uncovering content hidden inside items (e.g., when they open boxes). In that sense, and at the risk of losing the possibility to compare apes with humans, future tasks can adapt curiosity tasks into the apes’ environment, for example, by naturally covering and unveiling different food possibilities within their home range across time to cast exploration of new food sources. Relatedly, all our ape participants were adults. Future studies should thus aim to test comparable populations in terms of age, especially given young apes’ proclivity to explore relatively more at younger ages (see [49] for some preliminary results in this direction), to shed light on the comparative development of curiosity.

Second, future studies should explore in more detail children’s decisions in these scenarios. For example, one could present the intervention phase at the beginning of the task and then present trials where the visible rewards are better than those previously presented in the opaque cup during the initial phase. Will children reason that the same relation holds, or will they choose the conservative transparent option? Finally, given our finding that children did not necessarily maximize trials after discovering the content of the opaque cups, future curiosity studies may incorporate questionnaires after the cognitive task to shed light on the nature of their decisions.

In conclusion, our results suggest apparent species differences in the way children and apes deal with uncertainty. When a visible reward is available, apes tend not to explore the other option. Children, instead, are more curious and discover the content underneath the opaque cups faster than great apes—in particular, before the intervention phase. However, once great apes discover the hidden content, they often choose the opaque option, whereas children continue engaging in some level of exploration and diversify their options across trials.

## Supporting information

S1 File Supplementary materials file contains all the supporting tables and figures. (DOCX)

## References

[1] Heilbronner SR, Hayden BY. Contextual Factors Explain Risk-Seeking Preferences in Rhesus Monkeys. Front Neurosci. 2013;7. doi: 10.3389/fnins.2013.00007 23378827 PMC3561601

[2] Janmaat KRL, Ban SD, Boesch C. Chimpanzees use long-term spatial memory to monitor large fruit trees and remember feeding experiences across seasons. Anim Behav. 2013;86: 1183–1205.

[3] Janmaat KRL, Polansky L, Ban SD, Boesch C. Wild chimpanzees plan their breakfast time, type, and location. Proc Natl Acad Sci. 2014;111: 16343–16348. doi: 10.1073/pnas.1407524111 25349399 PMC4246305

[4] Paulsen D, Platt M, Huettel SA, Brannon EM. Decision-making under risk in children, adolescents, and young adults. Front Psychol. 2011;2: 72–72. doi: 10.3389/fpsyg.2011.00072 21687443 PMC3110498

[5] Haun DBM, Nawroth C, Call J. Great Apes’ Risk-Taking Strategies in a Decision Making Task. Santos L, editor. PLoS ONE. 2011;6: e28801. doi: 10.1371/journal.pone.0028801 22216113 PMC3244423

[6] Lakshminarayanan VR, Chen MK, Santos LR. The evolution of decision-making under risk: framing effects in monkey risk preferences. J Exp Soc Psychol. 2011;47: 689–693.

[7] Volz KG, Gigerenzer G. Cognitive processes in decisions under risk are not the same as in decisions under uncertainty. Front Neurosci. 2012;6: 105–105. doi: 10.3389/fnins.2012.00105 22807893 PMC3395005

[8] Rosati AG, Hare B. Chimpanzees and bonobos distinguish between risk and ambiguity. Biol Lett. 2010;7: 15–18. doi: 10.1098/rsbl.2010.0927 21106573 PMC3030905

[9] Kidd C, Hayden BY. The Psychology and Neuroscience of Curiosity. Neuron. 2015;88: 449–460. doi: 10.1016/j.neuron.2015.09.010 26539887 PMC4635443

[10] Berlyne DE. Novelty and curiosity as determinants of exploratory behaviour. Br J Psychol. 1950;41: 68–68.

[11] Berlyne DE. Conflict, arousal, and curiosity. 1960.

[12] Loewenstein G. The psychology of curiosity: A review and reinterpretation. Psychol Bull. 1994;116: 75–75.

[13] Gottlieb J, Oudeyer PY, Lopes M, Baranes A. Information-seeking, curiosity, and attention: computational and neural mechanisms. Trends Cogn Sci. 2013;17: 585–93. doi: 10.1016/j.tics.2013.09.001 24126129 PMC4193662

[14] Kang MJ, Hsu M, Krajbich IM, Loewenstein G, McClure SM, Wang JT, et al. The wick in the candle of learning: Epistemic curiosity activates reward circuitry and enhances memory. Psychol Sci. 2009;20: 963–973. doi: 10.1111/j.1467-9280.2009.02402.x 19619181

[15] Kidd C, Piantadosi ST, Aslin RN. The Goldilocks effect: Human infants allocate attention to visual sequences that are neither too simple nor too complex. PloS One. 2012;7: e36399. doi: 10.1371/journal.pone.0036399 22649492 PMC3359326

[16] Chu J, Schulz LE. Play, curiosity, and cognition. Annu Rev Dev Psychol. 2020;2.

[17] Mehlhorn K, Newell BR, Todd PM, Lee MD, Morgan K, Braithwaite VA, et al. Unpacking the exploration–exploitation tradeoff: A synthesis of human and animal literatures. Decision. 2015;2: 191–191.

[18] Sumner E, Steyvers M, Sarnecka BW. It’s not the treasure, it’s the hunt: Children are more explorative on an explore/exploit task than adults. 2019. pp. 2891–2897.

[19] Cohen JD, McClure SM, Yu AJ. Should I stay or should I go? How the human brain manages the trade-off between exploitation and exploration. Philos Trans R Soc B Biol Sci. 2007;362: 933–942. doi: 10.1098/rstb.2007.2098 17395573 PMC2430007

[20] Gopnik A. Childhood as a solution to explore–exploit tensions. Philos Trans R Soc B Biol Sci. 2020;375: 20190502. doi: 10.1098/rstb.2019.0502 32475327 PMC7293160

[21] Gopnik A, O’Grady S, Lucas CG, Griffiths TL, Wente A, Bridgers S, et al. Changes in cognitive flexibility and hypothesis search across human life history from childhood to adolescence to adulthood. Proc Natl Acad Sci. 2017;114: 7892–7899. doi: 10.1073/pnas.1700811114 28739917 PMC5544286

[22] Schulz E, Wu CM, Ruggeri A, Meder B. Searching for rewards like a child means less generalization and more directed exploration. Psychol Sci. 2019;30: 1561–1572. doi: 10.1177/0956797619863663 31652093

[23] Ruggeri A, Lombrozo T, Griffiths TL, Xu F. Sources of developmental change in the efficiency of information search. Dev Psychol. 2016;52: 2159. doi: 10.1037/dev0000240 27893251

[24] van Schaik CP, Pradhan GR, Tennie C. Teaching and curiosity: sequential drivers of cumulative cultural evolution in the hominin lineage. Behav Ecol Sociobiol. 2019;73: 2. doi: 10.1007/s00265-018-2610-7

[25] van Schaik CP, Burkart J, Damerius L, Forss SIF, Koops K, van Noordwijk MA, et al. The reluctant innovator: orangutans and the phylogeny of creativity. Philos Trans R Soc B Biol Sci. 2016;371: 20150183. doi: 10.1098/rstb.2015.0183 26926274 PMC4780526

[26] Forss SIF, Koski SE, van Schaik CP. Explaining the paradox of neophobic explorers: the social information hypothesis. Int J Primatol. 2017;38: 799–822.

[27] Damerius LA, Graber SM, Willems EP, van Schaik CP. Curiosity boosts orang-utan problem-solving ability. Anim Behav. 2017;134: 57–70.

[28] Murayama K, FitzGibbon L, Sakaki M. Process account of curiosity and interest: A reward-learning perspective. Educ Psychol Rev. 2019;31: 875–895.

[29] Byrne RW. Animal curiosity. Curr Biol. 2013;23: R469–R470. doi: 10.1016/j.cub.2013.02.058 23743408

[30] Glickman SE, Sroges RW. Curiosity in zoo animals. Behaviour. 1966;26: 151–187. doi: 10.1163/156853966x00074 5902289

[31] Oudeyer P-Y, Kaplan F. What is intrinsic motivation? A typology of computational approaches. Front Neurorobotics. 2009;1: 6–6. 10.3389/neuro.12.006.2007 PMC2533589 18958277

[32] Ryan RM, Deci EL. Intrinsic and extrinsic motivations: Classic definitions and new directions. Contemp Educ Psychol. 2000;25: 54–67. doi: 10.1006/ceps.1999.1020 10620381

[33] Gruber MJ, Gelman BD, Ranganath C. States of curiosity modulate hippocampus-dependent learning via the dopaminergic circuit. Neuron. 2014;84: 486–496. doi: 10.1016/j.neuron.2014.08.060 25284006 PMC4252494

[34] Wang MZ, Hayden BY. Monkeys are curious about counterfactual outcomes. Cognition. 2019;189: 1–10. doi: 10.1016/j.cognition.2019.03.009 30889493 PMC8029581

[35] Wu S, Blanchard T, Meschke E, Aslin RN, Hayden BY, Kidd C. Macaques preferentially attend to intermediately surprising information. Biol Lett. 2022;18: 20220144. doi: 10.1098/rsbl.2022.0144 35857891 PMC9256086

[36] Forss SIF, Motes-Rodrigo A, Dongre P, Mohr T, van de Waal E. Captivity and habituation to humans raise curiosity in vervet monkeys. Anim Cogn. 2022;25: 671–682. doi: 10.1007/s10071-021-01589-y 34855018 PMC9107434

[37] Forss S, Willems E. The curious case of great ape curiosity and how it is shaped by sociality. Ethology. 2022.

[38] Gentner D, Smith L. Analogical reasoning. Encycl Hum Behav. 2012;1: 130–136.

[39] Vlamings PHJM, Uher J, Call J. How the great apes (Pan troglodytes, Pongo pygmaeus, Pan paniscus, and Gorilla gorilla) perform on the reversed contingency task: The effects of food quantity and food visibility. J Exp Psychol Anim Behav Process. 2006;32: 60–60. doi: 10.1037/0097-7403.32.1.60 16435965

[40] Hanus D, Call J. Chimpanzee problem-solving: contrasting the use of causal and arbitrary cues. Anim Cogn. 2011;14: 871–878. doi: 10.1007/s10071-011-0421-6 21647648

[41] Call J. Inferences by exclusion in the great apes: the effect of age and species. Anim Cogn. 2006;9: 393–403. doi: 10.1007/s10071-006-0037-4 16924458

[42] Christie S, Gentner D, Call J, Haun DBM. Sensitivity to relational similarity and object similarity in apes and children. Curr Biol. 2016;26: 531–535. doi: 10.1016/j.cub.2015.12.054 26853364

[43] Meder B, Wu CM, Schulz E, Ruggeri A. Development of directed and random exploration in children. Dev Sci. 2021;24: e13095. doi: 10.1111/desc.13095 33539647

[44] De Petrillo F, Rosati AG. Variation in primate decision-making under uncertainty and the roots of human economic behaviour. Philos Trans R Soc B Biol Sci. 2021;376: 20190671. doi: 10.1098/rstb.2019.0671 33423637 PMC7815434

[45] Bohn M, Eckert J, Hanus D, Haun DBM. A longitudinal study of great ape cognition: Stability, reliability and the influence of individual characteristics. Proceedings of the Annual Meeting of the Cognitive Science Society. 2021.

[46] Boysen ST, Berntson GG, Mukobi KL. Size Matters: Impact of Item Size and Quantity on Array Choice by Chimpanzees (Pan troglodytes): 9. doi: 10.1037/0735-7036.115.1.106 11334213

[47] Beran MJ. The comparative science of “self-control”: what are we talking about? Front Psychol: 4. doi: 10.3389/fpsyg.2015.00051 25688226 PMC4311604

[48] Vlamings PHJM, Hare B, Call J. Reaching around barriers: the performance of the great apes and 3–5-year-old children. Anim Cogn. 2010;13: 273–285. doi: 10.1007/s10071-009-0265-5 19653018 PMC2822225

[49] Massen JJ, Antonides A, Arnold A-MK, Bionda T, Koski SE. A behavioral view on chimpanzee personality: Exploration tendency, persistence, boldness, and tool-orientation measured with group experiments. Am J Primatol. 2013;75: 947–958. doi: 10.1002/ajp.22159 23649750

