# Supplementary material for: Comparative curiosity: How do great apes and children deal with uncertainty?
Source: PLoS One. 2023 May 31;18(5):e0285946. doi: 10.1371/journal.pone.0285946 (PMC10231759; doi:10.1371/journal.pone.0285946)
Supplement: S1 File — (DOCX) [file pone.0285946.s001.docx]

**Comparative curiosity: how do great apes and children deal with uncertainty?**

Alejandro Sánchez-Amaro^1,2^, Federico Rossano^2^

^1^Departmant of Comparative Cultural Psychology, Max Planck Institute for Evolutionary Anthropology

^2^Department of Cognitive Science, University of California San Diego

**Supplementary Materials**

**Participants**

**Table S1: Information about all participants in Study 1-3.**

| **Subject** | **Specie** | **Year of birth** | **Sex** | **Participation in Study 1** | **Participation in test phase of Study 1** | **Participation in Study 2** | **Participation in test phase of Study 2** | **Participation in Study 3** |
| --- | --- | --- | --- | --- | --- | --- | --- | --- |
| Gemena | Bonobo | 2005 | female | Yes | No | Yes | Yes | Yes |
| Jasongo | Bonobo | 1990 | male | Yes | No | Yes | Yes | Yes |
| Kuno | Bonobo | 1996 | male | Yes | Yes | Yes | Yes | Yes |
| Lexi* | Bonobo | 1999 | female | Yes | No | Yes | No | No |
| Luiza | Bonobo | 2005 | female | Yes | Yes | Yes | No | No |
| Yasa | Bonobo | 1997 | female | Yes | No | Yes | Yes | Yes |
| Alex | Chimpanzee | 2001 | male | Yes | No | No | No | Yes |
| Corrie | Chimpanzee | 1976 | female | No | No | Yes | No | No |
| Daza | Chimpanzee | 1986 | female | No | No | No | No | Yes |
| Dorien | Chimpanzee | 1980 | female | Yes | Yes | Yes | No | No |
| Fraukje | Chimpanzee | 1976 | female | Yes | No | No | No | No |
| Frederike | Chimpanzee | 1974 | female | Yes | No | No | No | Yes |
| Frodo | Chimpanzee | 1993 | male | Yes | No | Yes | Yes | Yes |
| Hope | Chimpanzee | 1990 | female | Yes | No | No | No | Yes |
| Kisha | Chimpanzee | 2004 | female | Yes | No | No | No | No |
| Lobo | Chimpanzee | 2004 | male | Yes | Yes | Yes | Yes | Yes |
| Lome | Chimpanzee | 2001 | male | Yes | No | Yes | Yes | Yes |
| Natascha | Chimpanzee | 1980 | female | Yes | No | No | No | No |
| Robert | Chimpanzee | 1975 | male | Yes | Yes | Yes | Yes | Yes |
| Sandra | Chimpanzee | 1993 | female | Yes | Yes | Yes | Yes | Yes |
| Swela | Chimpanzee | 1995 | female | Yes | No | Yes | No | No |
| Tai | Chimpanzee | 2002 | female | Yes | No | Yes | Yes | Yes |
| Zira | Chimpanzee | 1997 | female | Yes | No | No | No | Yes |
| Diara | Gorilla | 2014 | female | Yes | No | No | No | No |
| Kibara | Gorilla | 2004 | female | Yes | No | No | No | No |
| Kumili | Gorilla | 2004 | female | Yes | Yes | No | No | No |
| Bimbo | Orangutan | 1980 | male | Yes | No | No | No | Yes |
| Dokana | Orangutan | 2002 | female | No | No | No | No | Yes |
| Padana | Orangutan | 1997 | female | Yes | Yes | No | No | Yes |
| Pini | Orangutan | 1988 | female | Yes | No | No | No | Yes |
| Raja | Orangutan | 2003 | female | Yes | No | No | No | Yes |
| Suaq | Orangutan | 2009 | male | Yes | No | No | No | Yes |

*The ape passed the criteria for participating in phases 2 and 3 of study 2. However, she did not participate due to an experimental error.

**Table S2: Percentage of times in which apes (studies 2 and 3) and humans (studies 4 and 5) choose the opaque cups in phases 1 and 3.**

| **Study** | **Phase 1** | **Phase 3** |
| --- | --- | --- |
| **2 (apes)** | 0% | 86% |
| **3 (apes)** | 16% | 68% |
| **4 (children)** | 52% | 77% |
| **5 (children)** | 41% | 56% |

**Model information**

Our five studies used R statistics (version 3.4.4) to fit GLMM models with binomial error structure. As an overall test of the eﬀects of our main predictors and potential interactions between them, in all our models we conducted a full-null model comparison (Forstmeier and Schielzeth, 2011) to avoid multiple testing, whereby the null model lacked the predictors of interest but was otherwise identical to the full model. This comparison was based on a likelihood ratio test (Dobson, 2002). We tested the eﬀect of individual ﬁxed eﬀects using likelihood ratio tests comparing the full model with reduced models lacking the ﬁxed eﬀects one at a time (Barr et al., 2013). Specifically, we investigated the significance of the test variables using the function drop1 from the lme4 package (Bates, 2010) to test each variable significance, including interactions between test predictors. Non-significant interactions were removed, and a new reduced model was fitted. A likelihood ratio test with significance set at p < 0.05 was used to compare models and to test the significance of the individual fixed effects. We ruled out collinearity by checking Variance Inflation Factors (VIF). All VIF values were closer to 1 (maximum VIF value = 1.07 across studies). We assessed the stability of our model by comparing the estimates derived by a model based on all data with those obtained from models with the levels of the random effects excluded one at a time. All models were stable. To obtain confidence intervals (CI) of the model estimates, we used a parametric bootstrap (function based on bootMer of the package lme4; N = 1000 bootstraps).

**Reliability of studies 1-5**

Two research assistants, unaware of the study hypothesis, coded a sub-sample of each study. The first reliability coder coded the sub-samples for study 1 (25% of the data), study 2 (20% of the data), study 3 (19% of the data) and study 5 (17.3% of the data). The second coder coded the sub-sample for study 4 (19.4% of the data). The reliability was excellent for the apes and children's choices in all our studies (lowest Cohen's Kappa score from study 5 = 0.98).

**Model information for study 1**

Our model investigated the likelihood of choosing the previous positive cup (PPC) during test sessions. That is the cup containing one single grape. In the model, we included the test variables type of trial, previous priming phase, and the two-way interaction between the two predictors. We included session and trial as control variables. We included individual ID and color ID as random effects and all the appropriate random slopes. The comparison between the full and the null model excluding the test predictors was significant (GLMM: *χ*^2^_3_ = 12.15, *p* = 0.007, N = 191). We dropped the non-significant two-way interaction between type of trial and previous priming phase (GLMM: *χ*^2^_1_ = 0.59, *p* = 0.44, N = 191). We found a main effect of the previous priming phase (GLMM: *χ*^2^_1_ = 10.77, *p* = 0.001, N = 191), suggesting that those individuals who experienced the positive priming were more likely to reject the previous positive cup in test and control trials (see Table S3).

Table S3: Model estimates for study 1.

| Term | Estimate | Standard Error | Chi-square | Degrees of freedom | p-value | Confidence interval |
| --- | --- | --- | --- | --- | --- | --- |
| Intercept | 7.43 | 2.21 | - | - | - | 3.07/26.26 |
| Type of trial (Test) | -1.17 | 1.34 | 0.67 | 1 | 0.41 | -10.57/2.25 |
| Priming (Positive) | -5.58 | 1.07 | 10.77 | 1 | **0.001** | -15.77/-1.59 |
| Session | 0.034 | 0.4 | 0.007 | 1 | 0.94 | -1.17/1.4 |
| Trial | 0.51 | 0.55 | 0.74 | 1 | 0.39 | -1.19/2.39 |
| Sex (male) | 0.41 | 0.94 | 0.14 | 1 | 0.71 | -2.41/12.71 |

**Model information for study 3**

Our model investigated the likelihood of choosing the opaque cup in phases 1 and 3 of our study. In consequence, the model included the test variable phase (phases 1 and 3) and species as our main effect. We included session, trial and the sex of the individual as control variables. We included individual ID and the location of the opaque cup as random effects and all the appropriate random slopes. The comparison between the full and the null model excluding the test predictors was significant (GLMM: *χ*^2^_3_ = 16.23, *p* = 0.001, N = 672). We found a non-significant effect of specie (GLMM; *χ*^2^_2_ = 2.57, *p* = 0.28, N = 672) and a main effect of phase (GLMM; *χ*^2^_1_ = 11.49, *p* = 0.001, N = 672) suggesting that great apes chose the opaque cup significantly more often in the third phase—after the intervention (see Table S4).

Table S4: Model estimates for study 3.

| Term | Estimate | Standard Error | Chi-square | Degrees of freedom | p-value | Confidence interval |
| --- | --- | --- | --- | --- | --- | --- |
| Intercept | -8.23 | 3.69 | - | - | - | -21.21/-3.04 |
| Phase (phase 3) | 10.76 | 2.41 | 11.49 | 1 | **0.001** | 7.17/22.79 |
| Specie (Chimpanzee) | 2.63 | 3.34 | 2.57 | 2 | 0.28 | 2.99/12.87 |
| Specie (Orangutan) | -1.77 | 3.64 | 2.57 | 2 | 0.28 | -12.78/7.22 |
| Session | 0.019 | 0.42 | 0.002 | 1 | 0.96 | -1.08/1.1 |
| Trial | 0.24 | 0.18 | 1.54 | 1 | 0.21 | -0.21/0.72 |
| Sex (male) | -0.88 | 2.35 | 0.18 | 1 | 0.68 | -5.95/3.6 |

**Model information for study 4**

Our model investigated children's likelihood of choosing the opaque cup in phases 1 and 3 of our study. Furthermore, we were also interested in developmental differences in children's choice behavior. In consequence, the model included the test variable phase (phases 1 and 3) and age as our main effect. We included trial and sex of the child as control variables. We included individual ID and the location of the opaque cup as random effects and all the appropriate random slopes. The comparison between the full and the null model excluding the test predictors was significant (GLMM; *χ*^2^_3_= 12.17, *p* = 0.007, N = 574). We found a main effect of phase (GLMM; *χ*^2^_1_= 7.43, *p* = 0.006, N = 574), suggesting that children chose the opaque cup significantly more often in phase 3. We also found a non-significant trend of age (GLMM; *χ*^2^_2_= 4.5, *p* = 0.1, N = 574), suggesting that older children were more likely to choose the opaque cup regardless of the study phase (see Table S4).

Table S5: Model estimates for study 4.

| Term | Estimate | Standard Error | Chi-square | Degrees of freedom | p-value | Confidence interval |
| --- | --- | --- | --- | --- | --- | --- |
| Intercept | 0.67 | 0.38 | - | - | - | -0.09/1.48 |
| Phase (phase 3) | 1.59 | 0.29 | 7.43 | 1 | **0.006** | 1.02/2.41 |
| Age (four) | -0.69 | 0.41 | 4.5 | 2 | 0.1 | -1.52/0.14 |
| Age (three) | -1.27 | 0.57 | 4.5 | 2 | 0.1 | -2.48/-0.12 |
| Trial | -0.09 | 0.11 | 0.53 | 1 | 0.17 | -0.35/ 0.15 |
| Sex (girl) | 0.2 | 0.31 | 0.35 | 1 | 0.88 | -0.47/ 0.81 |

**Model information for study 5**

Our model investigated children's likelihood of choosing the opaque cup in phases 1 and 3 of our study. As in study 4, we were also interested in developmental differences in children's choice behavior. In consequence, the model included the test variable phase (phase 1 and 3) as well as age as our main effect. We included trial and sex of the child as control variables. We included individual ID and the location of the opaque cup as random effects and all the appropriate random slopes. The comparison between the full and the null model excluding the test predictors was significant (GLMM; *χ*^2^_3_= 6.07, *p* = 0.048, N = 415). We found a main effect of phase (GLMM; *χ*^2^_1_= 4.42, *p* = 0.035, N = 574), suggesting that children chose the opaque cup significantly more often in phase 3 (see Table S6).

Table S6: Model estimates for study 5.

| Term | Estimate | Standard Error | Chi-square | Degrees of freedom | p-value | Confidence interval |
| --- | --- | --- | --- | --- | --- | --- |
| Intercept | -0.15 | 0.31 | - | - | - | -0.83/0.44 |
| Phase (phase 3) | 0.71 | 0.23 | 4.42 | 1 | **0.035** | 0.27/1.23 |
| Age (three) | -0.39 | -1.07 | 1.64 | 1 | 0.2 | -1.07/0.24 |
| Trial | 0.03 | 0.11 | 0.07 | 1 | 0.8 | -0.19/0.25 |
| Sex (girl) | -0.12 | 0.3 | 0.17 | 1 | 0.69 | -0.76/0.48 |

**Figure S1**

Figure S1: Bubbleplot depicting the number of times each child chose the opaque option in phases 1 and 3.





**Figure S2**


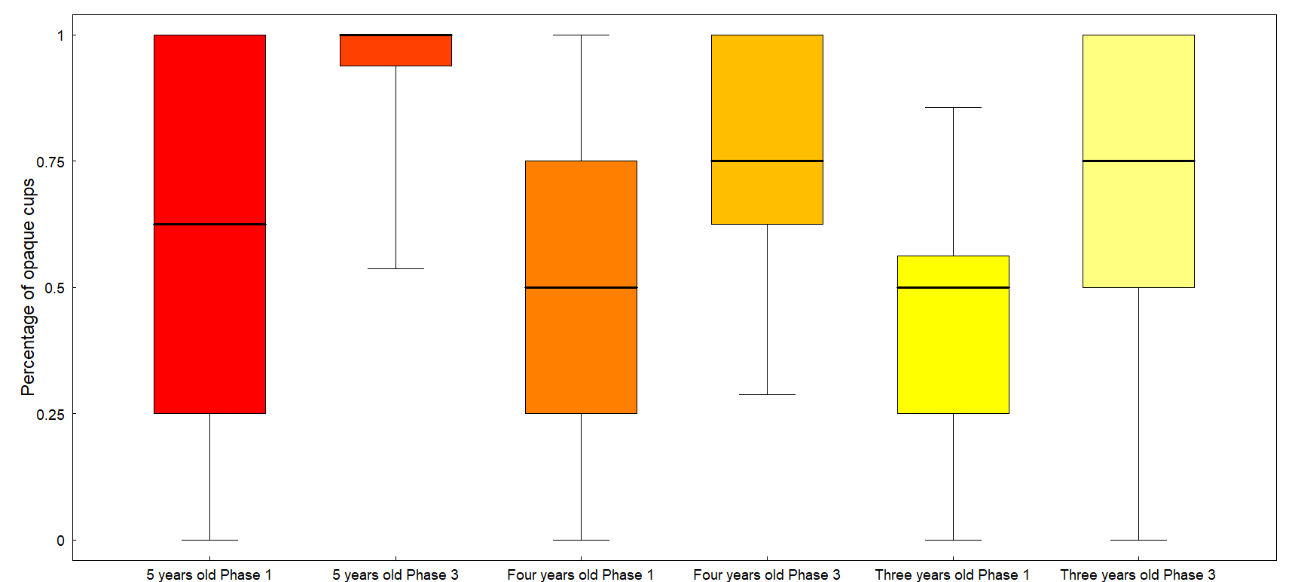


Figure S2: Percentage of trials in which children chose the opaque cup in Phases 1 and 3. The boxes represent the median and the interquartile range.

**Combination of study 3 and study 5 datasets**

Our model investigated whether children and apes differed in their likelihood of choosing the opaque cup between phases 1 and 3 of our studies 3 and 5. For this model we did treated species as “apes” and “human children” and we did not assess the age effects as in previous models for studies 4 and 5. In consequence, the model included the interaction of the test variables phase (phase 1 and 3) and species (apes and human children) as main effects. We included trial and sex of the the participants as control variables. We included individual ID and the location of the opaque cup as random effects and all the appropriate random slopes. The comparison between the full and the null model excluding the interaction was significant (GLMM; *χ*^2^_3_= 20.66, *p* = 0.0001, N = 1087). We found a main effect of the interaction between phase and specie (GLMM; *χ*^2^_1_= 8.72, *p* = 0.003, N = 1087), suggesting that while apes and children chose more often the opaque cup in the third phase, apes almost never chose the opaque cup in phase one while children already started to explore that option in the first study phase (see Table S6).

Table S6: Model estimates for combination of studies 3 and 5.

| Term | Estimate | Standard Error | Chi-square | Degrees of freedom | p-value | Confidence interval |
| --- | --- | --- | --- | --- | --- | --- |
| Intercept | -3.55 | 0.61 | - | - | - | -4.88/-2.42 |
| Condition*Specie | -4.04 | 0.77 | 8.72 | 1 | **0.003** | -5.79/-2.59 |
| Trial | 0.12 | 0.43 | 0.52 | 1 | 0.48 | -0.19/0.48 |
| Sex (male) | 0.02 | 0.77 | 0.003 | 1 | 0.96 | -0.87/0.88 |
